# Chronic Interstitial Nephritis

**Published:** 1896-10

**Authors:** J. A. Gillis

**Affiliations:** Buda, Texas


### TEXAS MEDICAL JOURNAL,

ESTABLISHED JULY, 1885.

PUBLISHED MONTHLY.—SUBSCRIPTION $2.00 A YEAR.

VOL. XII. AUSTIN, OCTOBER, 1896. NO. 4.

### Original Contributions.

For the Texas Medical Journal.

### CHRONIC INTERSTITIAL NEPHRITIS.

BY J. A. GILLIS, M. D., BUDA, TEXAS.

[Read before the Austin District Medical Society, June 21, 1896.]

CHRONIC INTERSTITIAL NEPHRITIS.—First, I will give a short anatomical and physiological description of the kidneys. I will not give the relational or minute anatomy only so far as will enable us to more fully comprehend the pathology, cause of symptoms and differential diagnosis between this and other forms of renal diseases.

I don't expect to advance anything new on the subject, but if this short paper will be a cause of making my fellow-practitioners, especially the country doctors, more thorough in their examinations, and in all obscure cases to always look to the kidneys before giving it up or sending the patient to our more favored brother in the city, I will feel amply repaid, for my experience has led me to believe that the country doctor, especially the older ones, rarely ever makes an urinalysis, or if he does at all it is only to apply the heat, and possibly Heller's tests, badly conducted, and if no albumen is found the patient is assured his kidneys are all right, and he is suffering with a simple dyspepsia, bronchitis, or some slight disorder which can be cured in a short time, but instead of relief the patient gets worse, his vision may be impaired, or a disturbance in the rhythm and force of the heart's action, and the unlooked for crisis appears as uremic convulsions, or coma, an apoplexia, a pneumonia, or some acute serous inflammation, which resists all treatment. Then the physician realizes his error; but alas! too late; and as the patient and friends have explicit faith in the doctor, no counsel is called, and soon the cold grave will hide the victim of his mistake.

The kidneys, generally two in number, are situated in the back part of the six upper regions of the abdominal cavity. They are imbedded in loose areolar tissues, and surrounded by a quantity of fat. They are about 4 inches long, 2½ broad, and something more than an inch in thickness. The normal weight is about 4½ to 6 ounces in the male, and 4 to 5½ in the female. While in this form of Bright's disease they may weigh only 2 ounces, or even less in extreme cases.

The kidney consists of a central cavity, the sinus, which is surrounded at all parts, except the hylum, by kidney substance proper, which is enveloped by a firm fibrous tissue, forming a close investment, which is easily stripped off, and leaves a smooth, even surface of a deep red color.

The kidney proper consists of an external cortical and an internal medullary portion. The cortical portion is about ⅓ to ½ an inch in thickness, and contains the malphigian bodies, which is the beginning of the tubuli uriniferi. The cortical portion undergoes the most change. Sometimes it is atrophied to 1-6 its normal thickness in this disease (Loomis').

Cysts, which are usually found in this disease, may be from a variety of causes, but are generally believed to be from inflammatory destruction of the normal cells which undergo colloid degeneration, fuse into a colloid mass, which is increased by successive layers, while at the same time new cells at the periphera become colloid. They are generally visible microscopically, but may be so small as to be detected only by the microscope.

The malphigian tufts may be obliterated by colloid cysts, but they generally atrophy, their capsule thickens, and connective tissue in abundance is deposited around them.

The adventitious connective tissue is sometimes deposited in the medullary portion also, and shrinking of the pyramids is the result, but generally it is not changed. In the advanced stage the capsule is greatly thickened, and there is more or less prolongation of the adventitious connective tissue from the capsule into the cortical substance, and then if the capsule is torn off the kidney surface will be ragged and uneven. Sometimes dilated veins are seen on the surface.

Etiology.—There are a great many factors in the cause of this disease, but the most potent are alcohol, syphilis and rheumatic or gouty diathesis. Added to these we have cold, lead poisoning and extensive atheromatous degeneration.

Alcohol causes three-fourths of all cases. More cases are caused by continual dram-drinking than by periodic drinking. And in this connection it is instructive to remember that while alcoholic drinks cause sclerosis and an increase in the connective tissue, malt liquors cause an abnormal deposit of fat, and fatty degeneration of the normal cells, and I think we, as a scientific profession, having the good of the people at heart, can not too strongly condemn their use, if only from the harm alcohol does in this disease, besides the other innumerable diseases and ills it is accountable for.

Syphilis is a great factor in the causation of this disease. Probably the reason that so many syphilitics suffer from organic kidney disease is because so few get proper treatment. Most of them will be very anxious until the primary lesion disappears, then, instead of taking a thorough eliminative course of treatment they will get careless, and finally stop altogether, until some troublesome secondary or severe tertiary lesion will drive them to the physician again.

It so frequently follows gout that it is often spoken of as the "gouty kidney," but I think it more likely that it is a secondary result associated with arterio sclerosis and extensive atheromatous degeneration.

Symptoms.—The symptoms of this form of Bright's disease are often obscure. There may be during life only vague nervous symptoms, while at the autopsy there may be found extensive cirrholic changes in the kidneys. The patient may grow weaker without any assignable cause. His disposition changes, he becomes morose and irritable. He may suffer from an almost continual headache, or repeated attacks of acute neuralgia, showing a deficient elimination of toxic elements from tissue metamorphosis, and consequently a deficiency in the nutrition of the nervous system. One of the earliest and most constant symptoms is a frequent desire to urinate, but it is so insidious in its approach it is hardly noticeable. The bladder may be emptied at late bed time, again the first thing after rising, and even to rise once during the night is not looked upon as being out of the ordinary for a man after the age of 40. There is no dropsy in one-fourth to one-half the cases, and if any at all only a slight œdema of the ankles or puffiness of the eyelids. In the latter stages there may be a grave discrasia of the fluids—there may be hemorrhagic infarctions. If there is a considerable dropsical effusion we can account for it by first, the changes in the urine that causes deterioration of the blood, which causes dropsy, and to further follow its effects we can easily see how dropsy in turn causes uremic poisoning, and uremia causes amblyopia, headache, violent inflammations, etc., which, if not relieved, will soon terminate in convulsions and coma, diarrhœa and vomiting, apoplexia, hydrothorax or pneumonia.

It usually runs an interrupted course. The symptoms will disappear and return with exacerbations, from cold, diet or regimen. During an acute exacerbation it will closely simulate acute nephritis. They are always worse after an exacerbation, hence we should be careful to diagnose the case early and give proper instructions as to diet, hygiene, etc., in order to avoid them. In the latter stages the features are pinched and sallow, the skin pale and anemic, but not so transparent as in chronic desquamative neprhritis. Often there is a marked cachexia. By careful examination we will nearly always find the force of the heart's action increased, and the second sound accentuated, caused by hypertrophy of the left ventricle, which in turn is a compensatory enlargement to overcome the increased blood pressure, especially in the kidneys.

Diagnosis.—In the earlier stages this form of Bright's disease is often difficult to diagnose, but if we remember certain leading features, and are thorough in our urinalysis, it is comparatively easy. First, we must remember that it almost always attacks one after the fortieth year, and is very insidious in its approach. By questioning closely we will nearly always get a history of alcoholism or syphilis. One-fourth of all cases are found in males. The urine is generally pale and abundant, and the low S. G. is characteristic, often ranging as low as 1005, but during an exacerbation it may be scanty, high-colored, and high S. G. The chemical tests are too numerous for me to mention in this paper. I usually try several, but principally rely on heat and the HNO3 contact tests. Ninety-five per cent. alcohol and trichloracetic acid are simple, and I believe very good.

If after a careful examination we get albumin but no casts we are not warranted in pronouncing it a cyclical, dietetic or physiological albuminuria, but must make repeated and careful microscopic examinations, and if we still find no casts or renal cells then we can assure ourselves and our patients that there is no organic disease of the kidney. While if casts be found we can, by close study, approximately estimate the extent of the damage by the number and kind of casts.

Casts are usually of the hyaline or hyalogranular variety, with some epithelium. Sometimes we can find fatty casts, and as the disease advances we may find blood, and in a few rare cases pus casts; and I will repeat, that by closely studying the kind of casts we can render a more accurate prognosis and institute a more scientific and satisfactory line of treatment.

In the absence of a microscope we have an invaluable aid in estimating the extent of injury to the kidneys by the administration of some of the balsams in small doses. Then we can roughly estimate how much of the kidneys are destroyed by the time it takes to get the odor and the quantity eliminated.

Treatment.—I have given no prognosis as in the course of time it will necessarily end fatally by some of its sequels or terminations, for the nature of the lesion does not admit of reparation.

It being incurable all lines of therapeutics must be utilized. Medical therapeutics are least called for primarily, but in the latter stages it may become necessary to administer the most active drugs to avert immediate death.

Primarily we must look to the diet and general hygienic surroundings. The diet should be light, easily digested, and of the most nutritious aud assimilable variety. Vegetable tonics and artificial digestants should be used to keep the health up to the highest possible standard. All drinks containing alcohol in any form should be prohibited. The clothing should be sufficient to protect the patient from sudden changes. Flannel should be worn next to the skin at all seasons. An ordinary warm bath daily with a hot bath once a week will keep the skin in good condition, which is very necessary. The bowels should be kept soluble by the alternate use of salines and vegetable laxatives. While in the latter stages, if there should be an excessive dropsical effusion, we may use a drastic purgative, as elaterium or com. jalap powder, or we can give large and repeated doses of salts (Mg. $SO_4$), which is simple, and in my judgment the most efficient.

Stimulating diuretics must not be used. Digitalis, I think, will ordinarily meet all requirements, while in extreme cases, as uremic coma, or where uremic intoxication is threatened, we can use F. E. pilocarpus in full doses, hot packs, etc. Loomis advises the hypodermic use of morphia in sufficient doses to relieve and prevent muscular spasms, and by so doing the secretions will be re-established, and I have seen apocynum, in small doses, highly recommended when the symptoms were not urgent, but I have never used either of them.

As my experience has been limited both in practice and in writing papers, I will leave the subject to be discussed by the society, and I think if discussed according to the merits, not of the paper, but the subject, it will benefit us all. I am sure it will be beneficial to me.

